# Strategies for prevention of gastrointestinal cancers in developing countries: a systematic review

**DOI:** 10.7189/jogh.07.020405

**Published:** 2017-12

**Authors:** Ahmad Zia Shams, Ulrike Haug

**Affiliations:** 1 Epidemiological Cancer Registry Baden–Wuerttemberg, German Cancer Research Centre, Heidelberg, Germany; 2 Department of Clinical Epidemiology, Leibniz Institute for Prevention Research and Epidemiology, Bremen, Germany; 3 Faculty of Human and Health Sciences, University of Bremen, Bremen, Germany

## Abstract

**Background:**

Gastrointestinal cancers account for one third of total cancer incidence and mortality in developing countries. To date, there is no systematic synthesis of evidence regarding strategies to prevent gastrointestinal cancers in developing countries. We aimed to provide a systematic overview of studies evaluating strategies for prevention or early detection of the three most common gastrointestinal cancers (gastric, liver and colorectal cancer) in developing countries.

**Methods:**

We searched MEDLINE, Web of Science and WHO Global Index Medicus databases for relevant articles published until October 2016 using combinations of the search terms “gastrointestinal”, “digestive system”, “gastric”, “liver”, “colorectal”, “cancer”, “prevention”, “early detection” and “developing country” (including names).

**Results:**

Overall, 73 articles met the inclusion criteria, providing information on short– and long–term outcomes (up to 30 years) from various intervention studies (∼45% randomized). Trials on hepatitis B vaccination consistently showed vaccine efficacy over time and indicated long–term preventive effects on liver cancer incidence that start to become measurable at the population level. Studies on anti–*H. pylori* treatment suggested a reduction in gastric cancer incidence reaching statistical significance after long–term follow–up, while evidence regarding a preventive effect in persons with precancerous lesions is still inconclusive. The studies regarding colorectal cancer focused on early detection, ∼90% of which were restricted to intermediate endpoints.

**Conclusion:**

In conclusion, there were a number of studies on gastric and liver cancer prevention in developing countries showing promising results after long–term follow–up. Important next steps include pooled meta–analyses as far as possible given the heterogeneity between studies as well as implementation research.

Cancer is a leading health burden and cause of death worldwide, with approximately 14 million new cases and 8 million deaths per year globally [1]. Despite the general understanding that cancer is primarily a problem of the (industrially) developed world, more than 60% of cancer cases and 70% of cancer deaths were estimated to occur in developing countries in 2008 [2]. Gastrointestinal cancers (GICs) are estimated to account for one third of total cancer incidence and mortality in developing countries [1]. With almost 2 million new cases and 1.5 million deaths in 2012, gastric, liver and colorectal cancers (CRC) are currently estimated to be the three most common GICs in the less developed regions of the world, where they account for 24%, 23% and 22% of all GICs, respectively [1].

Despite extensive efforts to improve treatment of metastatic disease including the development of new drugs, the prognosis for advanced stages of gastric, liver and colorectal cancer remains poor even in developed countries, with 5–year relative survival rates (regional and distant stages combined) of 33%, 8% and 49% in the US in 2016, respectively [3]. These figures highlight the need for further efforts to realize the high potential of primary prevention and early detection of these GICs. This is particularly relevant for developing countries where up–to–date treatment of advanced stage cancers may additionally be limited due to infrastructure and economic constraints.

To date there has been no systematic review regarding strategies to prevent these GICs in developing countries. We therefore plsaimed to conduct a systematic literature search and provide an overview of studies evaluating strategies for prevention or early detection of the three most common gastrointestinal cancers (gastric, liver and colorectal cancer) in developing countries.

## METHODS

### Search strategy

We searched MEDLINE, Web of Science and WHO Global Index Medicus databases for articles published until October 2016. We used a comprehensive search strategy with no restriction regarding publication date, type of participants (eg, age or sex characteristics), type of interventions, study design or type of outcome measures. A detailed description of our search strategy is provided in Appendix S1 in **Online supplementary document(Online Supplementary Document)**. In brief, we used both free text keywords and for the MEDLINE search also MeSH (Medical Subject Heading) terms. Regarding the latter, we used the MeSH terms gastrointestinal cancer, digestive system cancer (entailing gastric, colorectal and liver cancer as MeSH sub–categories), prevention, early detection of cancer, and developing country, as search terms.

We used the United Nations Development Programme (UNDP) country classification (for the year 2013), which utilizes Human Development Index (HDI) as basis of country groupings, for determining countries with “developing” status. The classification groups all countries in very high, high, medium and low HDI clusters. Countries with high, medium and low HDI are classified as developing countries. Consequently, the names of 140 developing countries were also used as search terms. The names of these countries are listed in Appendix S2 in **Online Supplementary Document(Online Supplementary Document)**. In addition to the database searches, we employed cross–referencing to complement the study identification process. Duplicate publications were deleted. In a first step, each title and abstract was screened, to determine whether the article was potentially relevant for the review topic. In a next step, the full text of potentially relevant articles was reviewed to assess whether inclusion criteria were fulfilled. This was done independently by both authors.

### Inclusion and exclusion criteria

We included studies that aimed at evaluating strategies for prevention of gastric, liver and colorectal cancer in developing countries. We only included studies on humans published in English. We focused on studies that reported disease–related outcomes such as long–term health outcomes (eg, reduction of incidence or mortality) or intermediate outcomes that are expected to be associated with long–term effects (eg, detection rates or vaccine efficacy). Accordingly, we excluded non–original articles (eg, case reports, commentaries, guidelines etc.) and studies that were restricted to health behavior.

### Data extraction

All studies meeting the inclusion criteria were categorized by cancer type (gastric cancer, liver cancer, colorectal cancer). Within these categories, we ordered the studies by the type of intervention or preventive strategy. We extracted the following information in a standardized manner from all included studies: author, publication year, preventive measure including details such as the intensity and length of interventions or the number of screening rounds, country and region within the country, study design, study population (sample size, sex distribution and age), outcomes under study and results. If the same study population was examined at different time points after the intervention, we extracted the information on the outcomes under study and results for each follow–up. Both authors reviewed the articles independently and any disagreement was resolved by consensus.

We applied the Preferred Reporting Items for Systematic Reviews and Meta–Analyses (PRISMA) guidelines criteria as far as it was possible, given the heterogeneity of the studies. The PRISMA Checklist is provided in Appendix S3 in **Online Supplementary Document(Online Supplementary Document)**.

## RESULTS

**Figure 1:**
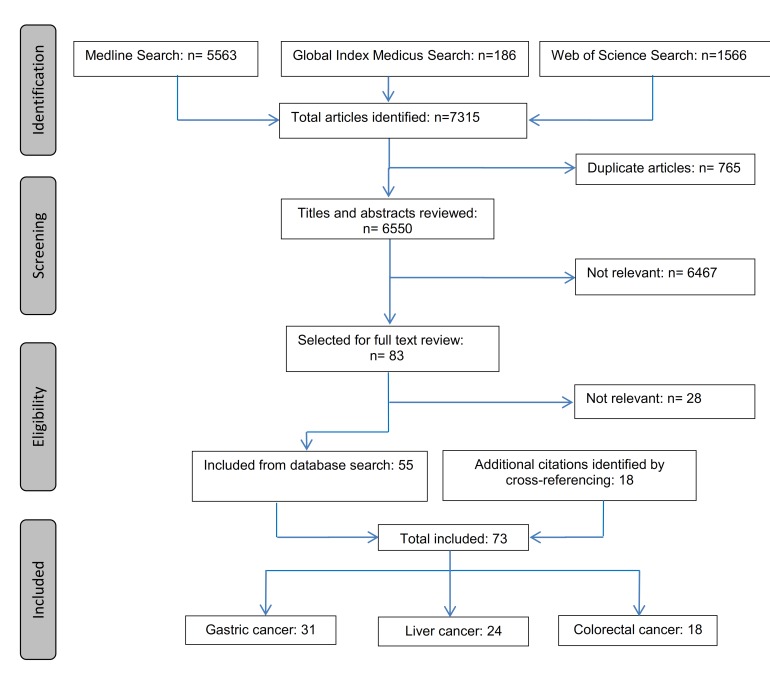
Flow diagram of the literature search process.

Overall, our initial search yielded 7315 entries. After deleting duplicates (n = 765) and excluding articles that were not relevant to the topic according to their title and abstract (n = 6467), 83 articles were selected for the full text review. Of these, 55 articles were relevant. Another 18 relevant articles were identified by cross–referencing, yielding in total 73 articles (**Figure 1**). The underlying number of studies is lower (n = 54) because several articles refer to the same study but report on different follow–up periods or outcomes.

### Gastric cancer

Studies on gastric cancer prevention could be classified into three main categories: supplementation with vitamins and minerals (4 randomized trials), anti–*H. pylori* treatment (12 randomized trials) and early detection with an occult blood bead detector, with photofluorography, with X–ray or direct gastroscopy (5 cross–sectional diagnostic studies, 2 case–control studies and 1 non–randomized intervention trial). **Table 1** provides detailed information about these studies [4–34].

**Table 1:** Studies investigating strategies for prevention of gastric cancer

Author(s), year	Preventive measure/screening tool	Country (region)	Study design	Study population	Outcome(s) under study	Results	
**Supplementation with vitamins and minerals**							
Blot et al., 1993 [4]; Wang et al., 1994 [5]; Qiao et al., 2009 [6]	Daily supplementation of: Factor A (retinol + zinc); Factor B (riboflavin + niacin); Factor C (vitamin C+molybdenum); Factor D (beta–carotene + vitamin E+selenium); Duration of supplementation: 5.25 y	China (Linxian)	Randomized trial with 2^4^ factorial design	Sample size: n = 29 584; Sex distribution: 45% male; Age: range: 40–69 y	RR regarding reduction of gastric cancer incidence and mortality determined at the end of the intervention period; OR regarding reduction of prevalence of gastric dysplasia and cancer determined by endoscopic evaluation at the end of the intervention period; HR regarding reduction of gastric cancer mortality determined at 15 y follow–up	**RR regarding gastric cancer incidence:**	**RR regarding gastric cancer mortality:**
						Factor A: 0.96 (95% CI 0.81–1.14)	Factor A: 1.03 (95% CI 0.83–1.28)
						Factor B: 1.04 (95% CI 0.88–1.23)	Factor B: 1.00 (95% CI 0.81–1.24)
						Factor C: 1.10 (95% CI 0.92–1.30)	Factor C: 1.09 (95% CI 0.88–1.36)
						Factor D: 0.84 (95% CI 0.71–1.00)	Factor D: 0.79 (95% CI 0.64–0.99)
						**OR regarding dysplasia or cancer:**	**OR regarding gastric cancer:**
						Factor A: 0.58 (95% CI 0.24–1.39)	Factor A: 0.38 (95% CI 0.13–1.15)
						Factor B: 1.32 (95% CI 0.56–3.14)	Factor B: 1.67 (95% CI 0.58–4.76)
						Factor C: 2.64 (95% CI 1.01–6.93)	Factor C: 2.75 (95% CI 0.86–8.84)
						Factor D: 0.83 (95% CI 0.35–2.01)	Factor D: 1.05 (95% CI 0.37–2.92)
						**HR regarding gastric cancer mortality:**	
						Factor A: 0.97 (95% CI 0.87–1.09)	
						Factor B: 0.90 (95% CI 0.88–1.10)	
						Factor C: 1.05 (95% CI 0.94–1.18)	
						Factor D: 0.89 (95% CI 0.79–1.00)	
Li et al., 1993 [7], Dawsey et al., 1994 [8], Wang et al., 2013 [9]	Daily supplementation of: 14 vitamins and 12 minerals; Duration of supplementation: 6 y	China (Linxian)	Randomized two–armed placebo–controlled trial	Sample size: n = 3318 (adults with cytologically detected oesophageal dysplasia); Sex distribution: 44% male; Age: median: 54 y	RR regarding reduction of gastric cancer incidence and mortality; OR regarding reduction in the prevalence of gastric dysplasia and cancer determined by endoscopic evaluation at 2.5 and 6 y follow–up; HR regarding reduction of gastric cancer mortality determined at 26 y follow–up	**RR regarding gastric cancer incidence:**	**RR regarding gastric cancer mortality:**
						1.17 (95% CI 0.87–1.58)	1.18 (95% CI 0.76–1.85)
						**Lesion prevalence at 2.5 y:**	**Lesion prevalence at 6 y:**
						Dysplasia or cancer:	Dysplasia or cancer:
						OR: 2.49 (95% CI 0.94–6.58)	OR: 0.77 (95% CI 0.41–1.47)
						Gastric cancer:	Gastric cancer:
						OR: 1.91 (95% CI 0.64–5.68).	OR: 0.77 (95% CI 0.38–1.58)
						**HR regarding gastric cancer mortality:**	
						0.91 (95% CI 0.73–1.13)	
Li et al., 2004 [10]	Supplementation of: allitridum (daily) and selenium (every other day); Duration of supplementation: 2 months supplementation in 2 y	China (Shandong)	Randomized two–armed placebo– controlled trial	Sample size: n = 5033; Sex distribution: 65% male; Age: range: 35–74 y	RR regarding gastric cancer determined at 5 y follow–up	RR: 0.48 (95% CI 0.21–1.06) Subgroup analysis restricted to males: RR: 0.36 (95% CI 0.14–0.92)	
Plummer et al., 2007 [11]	Daily supplementation of: vitamin C, vitamin E and beta carotene; Duration of supplementation:3 y	Venezuela (Tashira)	Randomized two–armed placebo–controlled trial	Sample size: n = 1980; Sex distribution: 47% male; Age: range: 35–69 y	RR regarding regression of precancerous lesions	RR:1.02 (95% CI 0.90–1.33)	
**Anti *H. pylori* treatment**							
Correa et al., 2000 [12]	Anti–*H. pylori* treatment with: amoxicillin, metronidazole and bismuth subsalicylate; Duration of treatment: 2 weeks; Daily supplementation of: beta–carotene and/or ascorbic acid; Duration of supplementation: 6 y	Colombia (Narino)	Randomized placebo– controlled trial with 2^3^ factorial design	Sample size: n = 852 (individuals with precancerous lesions – multi–focal atrophy and/or intestinal metaplasia); Sex distribution: 46% male; Age: mean: 51 y	RR regarding regression of precancerous lesions at 7 y follow–up	**Multi–focal atrophy:**	
						Anti–*H. pylori* treatment:	
						RR: 4.80 (95% CI 1.60–14.20)	
						Beta–carotene:	
						RR: 5.1 (95% CI 1.70–15.00)	
						Ascorbic acid:	
						RR: 5.00 (95%CI 1.70–14.40)	
						**Intestinal metaplasia:**	
						Anti–*H. pylori* treatment:	
						RR: 3.10 (95% CI 1.00–9.30)	
						Beta–carotene:	
						RR: 3.40 (95% CI 1.10–9.80)	
						Ascorbic acid:	
						RR: 3.30 (95% CI 1.10–9.50)	
Sung et al., 2000 [13]; Zhou et al., 2014 [14]	Anti–*H. pylori* treatment with: omeprazole, amoxicillin and clarithromycin; Duration of treatment: 1 week	China (Yanati)	Randomized two–armed placebo–controlled trial	Sample size: n = 587 (*H. pylori* positive); Sex distribution: 50% male (treatment group), 46% male (placebo group); Age: mean: 50 y (treatment group), 51 y (placebo group)	Changes in histologic grading: determined at 1 y follow–up; determined at 10 y follow–up	**Regression of gastric atrophy: **(*P* = 0.94)	
						**Regression of intestinal metaplasia: **(*P* = 0.52)	
						**Regression of gastric atrophy:**	
						RR: 0.88 (95% CI 0.79–0.97) in antrum	
						RR: 0.62 (95% CI 0.49–0.77) in corpus	
						**Regression of intestinal metaplasia:**	
						RR: 0.85 (95% CI 0.78–0.92) in antrum	
						RR: 0.87 (95% CI 0.74–1.02) in corpus	
						**Regression of atypical dysplasia:**	
						RR: 1.33 (95% CI 0.85–2.07) in antrum	
						RR: 1.01 (95% CI 0.38–2.68) in corpus	
Guo et al., 2003 [15]	Health education: both intervention and control group; Treatment of high risk subjects (with precancerous lesions): antibiotics, Chinese herb medicine and nutritional therapy (only intervention group)	China (Zhuanghe)	Cluster–randomized two–armed controlled intervention study	Sample size: n = 100 966 (of which n = 1781 were identified as high risk subjects); Sex distribution: 51% male (intervention group), 50% male (control group); Age: >35 y	RR regarding gastric cancer mortality at 3 y follow–up	RR: 0.50 (95% CI 0.34–0.73)	
Zhou et al., 2003 [16]	Anti–*H. pylori* treatment with: omeprazole, clarithromycin and amoxicillin; Duration of treatment: 1 week	China (Shandong)	Randomized two–armed placebo– controlled trial	Sample size: n = 552 (*H. pylori* positive); Sex distribution: n.r.; Age: range: 35–75 y	Proportion of subjects in whom severity of precancerous lesions has improved/not changed since baseline in *H .pylori*–positive vs *H. pylori* negative subjects at 5 y follow–up	Only for intestinal metaplasia in antrum the proportion of subjects in whom lesion severity has improved/not changed was higher in *H. pylori* negative vs *H. pylori* positive subjects: 71% vs 61% (*P* = 0.032). For other lesions in the antrum and for lesions in the body there were no significant differences in the proportions.	
Wong et al., 2004 [17]	Anti–*H. pylori* treatment with: omeprazole, amoxicillin, clavulanate potassium and metronidazole; Duration of treatment: 2 weeks	China (Fujian)	Randomized two–armed placebo–controlled trial	Sample size: n = 1630 (healthy carriers of *H. pylori*); Sex distribution: 54% male; Age: mean: 42 y	HR regarding gastric cancer incidence at 7.5 y follow–up; Subgroup analysis restricted to subjects without precancerous lesions	HR: 0.63 (95% CI 0.24–1.62) n = 0 in treatment group vs n = 6 in control group (*P* = 0.02)	
Ley et al., 2004 [18]	Anti–*H. pylori* treatment with: omeprazole, amoxicillin and clarithromycin; Duration of treatment: 1 week	Mexico (Chiapas)	Randomized two–armed placebo–controlled trial	Sample size: n = 248 (healthy carriers of *H. pylori*); Sex distribution: 37% male; Age: mean: 51 y (treatment group), 52 y (control group)	*H. pylori* cure rate at 6 weeks and 1 y follow–up; Changes in worst biopsy diagnosis at 6 weeks and 1 y follow–up	Cure rate: 6 weeks: 79% (treatment) vs 3% (placebo) (*P* < 0.001) 1 y: 76% (treatment) vs 2% (placebo) (*P* < 0.001) No difference regarding the change in worst biopsy diagnosis between groups.	
You et al., 2006 [19]; Ma et al., 2012 [20], Li et al., 2014 [21]	Anti–*H. pylori* treatment / supplementation in *H. pylori* seropositives with: omeprazole, amoxicillin and/or garlic supplements and/or vitamins/minerals (vitamin C, vitamin E and selenium); Duration of treatment: 2 weeks; Supplementation in *H. pylori* seronegatives with: garlic and/or vitamin C, vitamin E and selenium; Duration of supplementation: 7.3 y	China (Shandong)	Randomized placebo–controlled trial with 2^3^ and 2^2^ factorial design	Sample size: n = 3365; *H. pylori* seropositives: n = 2258; *H. pylori* seronegatives: n = 1107 (all underwent gastroscopy at baseline); Sex distribution: 51% male; Age: mean: 47 y, range: 35–64 y	OR regarding reduction in the prevalence of advanced precancerous lesions determined at 3.5 and 7.5 y follow–up; OR regarding gastric cancer incidence and HR regarding gastric cancer mortality determined at 15 y follow–up; Subgroup analysis in subjects ≥55 y (results refer to anti–*H. pylori* treatment).	**Prevalence of advanced precancerous lesions:**	
						Anti–*H. pylori* treatment:	
						3.5 y: OR: 0.77 (95% CI 0.62–0.95)	
						7.5 y: OR = 0.60 (95% CI 0.47–0.75)	
						Garlic:	
						3.5 y: OR = 0.9 (95% CI 0.84–1.18)	
						7.5 y: OR = 1.08 (95% CI 0.90–1.29)	
						Vitamins/minerals:	
						3.5 y: OR = 1.32 (95% CI 1.12–1.57)	
						7.5 y: OR = 1.14 (95% CI 0.96–1.37)	
						**Gastric cancer incidence / Gastric cancer mortality:**	
						Anti–*H. pylori* treatment:	
						OR: 0.61 (95% CI 0.38–0.96) / HR: 0.67 (95% CI 0.36–1.28)	
						Garlic:	
						OR: 0.80 (95% CI 0.53–1.20) / HR: 0.65 (95% CI 0.35–1.20)	
						Vitamins/minerals	
						OR: 0.81 (95% CI 0.54–1.22) / HR: 0.55 (95% CI 0.29–1.03)	
						**Gastric cancer incidence / Gastric cancer mortality**	
						OR: 0.36 (95% CI 0.17–0.79) / HR: 0.26 (95% CI 0.09–0.79)	
						**Gastric cancer incidence among subjects with intestinal metaplasia and dysplasia:**	
						OR: 0.56 (95% CI 0.34–0.91)	
Ji et al., 2006 [22]	Anti–*H. pylori* treatment with: omeprazole/lansoprazole, clarithromycin, bismuth citrate and tinidazole; Duration of treatment: 2 weeks	China (Zhejiang)	Randomized two–armed placebo– controlled trial	Sample size: n = 48 (with hyperplastic gastric polyps and *H. pylori*^+^); Sex distribution: 54% male; Age: range: 21–73 y; mean: 49 y (treatment arm), 47 y (control arm)	*H. pylori* cure rate; Polyp disappearance rate determined at 1 y follow–up	Treatment arm: 86% (95% CI 63%–99%); Control arm: 0% (95% CI 0%–21%) Treatment arm: 68% (95% CI 54%–91%); Control arm: 0% (95% CI 0%–21%)	
Sari et al., 2008 [23]	Anti–*H. pylori* treatment with: clarithromycin, amoxicillin and proton pump inhibitor; Duration of treatment: 2 weeks	Turkey	Randomized two–armed intervention study	Sample size: Group 1: n = 70 (*H. pylori*^+ ^index patients and their *H. pylori*^+ ^family members); Group 2: n = 70 (only *H. pylori*^+ ^index patients treated); Sex distribution: n.r.; Age: mean: 42 y	Rate of *H. pylori* positives determined at 9 month follow–up	Group 1: 7%; Group 2: 39%; OR: 8.61 (95% CI 2.91–22.84)	
Wong et al., 2012 [24]	Anti–*H. pylori* treatment with: omeprazole, amoxicillin and clarithromycin followed by celecoxib; Duration of treatment: Anti–*H. pylori*: 1 week; Celecoxib: 2 y	China (Linqu)	Randomized placebo–controlled trial with 2^2^ factorial design	Sample size: n = 1024 (*H. pylori*^+ ^patients with advanced PLs); Sex distribution: 46% male; Age: range: 35–64 y	OR regarding regression of advanced PLs determined at 2 y follow–up	**Regression of advanced PLs:** Anti–*H. pylori* treatment: OR: 1.80 (*P* = 0.009); Celecoxib: OR: 1.55 (*P* = 0.04); Anti–*H. pylori* treatment + celecoxib: OR: 1.50 (*P* = 0.067)	
Massarrat et al., 2012 [25]	Anti–*H. pylori* treatment with: bismuth subcitrate, metronidazole and furazolidone; Duration of treatment: 2 weeks	Iran (Tehran)	Randomized two–armed placebo– controlled trial	Sample size: n = 521 (*H. pylori*^+ ^1^st^ degree relatives of gastric cancer patients); Sex distribution: 49% male; Age: mean: 48 y, range: 38–70 y	Proportion of subjects in whom severity of PLs changed by at least one score in the treatment group vs the control group determined at 2.5 and 4.5 y follow–up	**Atrophy in antrum:**	
						2.5 y: 62% vs 31% (*P* < 0.0001)	
						4.5 y: 50% vs 30% (*P* > 0.05)	
						**Atrophy in corpus:**	
						2.5 y: 36% vs 13% (*P* < 0.001)	
						4.5 y: 43% vs 21% (*P* < 0.02)	
						**Intestinal metaplasia in antrum:**	
						2.5 y: 35% vs 28% (*P* > 0.05)	
						4.5 y: 38% vs 21% (*P* > 0.05)	
						**Intestinal metaplasia in corpus:**	
						2.5 y: 9% vs 10% (*P* > 0.05)	
						4.5 y: 20% vs 29% (*P* > 0.05)	
Pan et al., 2016 [26]	Anti–*H. pylori* treatment with: high dose of tetracycline, metronidazole, omeprazole and bismuth citrate (group 1) or placebos of tetracycline and metronidazole plus low dose of omeprazole and bismuth citrate (group 2)	China (Linqu)	Cluster–randomized two–armed placebo–controlled trial	Sample size: group 1: n = 44 345; group 2: n = 43 930 (all *H. pylori* positive); Sex distribution: 42% male; Age: range: 25–54 y; median:43 y	*H. pylori* cure rate assessed 45 days after treatment	Group 1: 73%; Group 2: 15%	
**Early detection**							
Qin et al., 1988 [27]	Occult blood bead detector; positive results followed up by gastroscopy	China (Henan & Jiangsu)	Cross–sectional diagnostic study	Sample size: n = 38 073; Sex distribution: 42% male; Age: range: 35–70 y	Positivity rate; Gastric cancer detection rate; Proportion of gastric cancers detected at an early stage	24% (9204/38 073); 0.2% regarding the whole study population (85/38 073); 2% regarding those who underwent gastroscopy (85/4023); 45% (57/126)	
Qin et al., 1997 [28]	Occult blood bead detector; positive results followed up by gastroscopy	China (Henan)	Cross–sectional diagnostic study	Sample size: n = 4970; Sex distribution: n.r.; Age: range: 30–70 y	Positivity rate; Gastric cancer detection rate; Proportion of gastric cancers detected at an early stage	7% (372/4,970); 0.2% regarding the whole study population (11/4,970); 1% regarding those who underwent gastroscopy (11/817); 84% (9/11)	
Pisani et al., 1994 [29]	Photofluorography	Venezuela (Tashira)	Case–control study	Sample size:n = 241 (cases), n = 2410 (controls); Sex distribution: n.r.; Age: ≥35 y	OR regarding reduction in gastric cancer mortality	OR: 1.26 (95% CI 0.83–1.91)	
Rosero–Bixby et al., 2007 [30]	X–ray	Costa Rica (Cartago and Perez Zeledon)	Non–randomized community–controlled study (measures before and after intervention)	Sample size: n = 6828; Sex distribution: 64% male; Age: mean: 64 y	Gastric cancer death rate at 2–7 y follow–up in the intervention group vs four control groups	Reduction in death rate by 48–59% (*P* < 0.05)	
Zhang et al., 2002 [31]	Direct gastroscopy	China	Cross–sectional diagnostic study	Sample size: n = 3048; Sex distribution: 95% male; Age: range 60–93 y, mean: 70 y	Gastric cancer detection rate; Proportion of gastric cancers detected at an early stage	3% (92/3048); 63% (58/92)	
Lu et al., 2014 [32]	Direct gastroscopy	China (Henan)	Cross–sectional diagnostic study	Sample size: n = 36 154; Sex distribution: 59% male; Age: range 40–69 y	Gastric cancer detection rate; Proportion of gastric cancers detected at an early stage	0.84% (307/36154); 79% (243/307)	
Zheng et al., 2015 [33]	Direct gastroscopy	China (Yangzhong)	Cross–sectional diagnostic study	Sample size: n = 12 453; Sex distribution: 43% male; Age: range: 40–69 y	Gastric cancer detection rate (mucosal and submucosal carcinoma and high–grade intraepithelial neoplasia) Proportion of gastric cancers detected at an early stage	0.48% (60/12 453); excluding high–grade intraepithelial neoplasia: 0.37% (47/12 453); 100% (60/60)	
Chen et al., 2016 [34]	Direct gastroscopy	China (Linzhou)	Case–control study	Sample size: cases: n = 313 (individuals who died of gastric cancer), controls: n = 1876; Sex distribution: 69% male; Age: range: 40–69 y	OR regarding reduction in gastric cancer mortality	OR: 0.72 (95% CI 0.54–0.97)	

HR – hazard ratio, n – number, n.r. – not reported, OR – odds ratio, PLs – precancerous lesions, RR – relative risk, y – year

Studies on vitamin and mineral supplementation (eg, vitamin A, B vitamins, selenium etc.) considered reduction in gastric cancer incidence and mortality [4,6,7,9,10] or changes regarding precancerous lesions as outcomes [5,8,11]. All these studies were conducted in China except one [11]. The duration of supplementations ranged between 2–6 years and follow–up periods between 5 and 26 years.

The supplementation of a combination of beta–carotene, vitamin E and selenium showed a (marginally) statistically significant reduction in gastric cancer mortality of 20% (relative risk (RR) = 0.79, 95% confidence interval (CI) 0.64–0.99) [4]. Other studies, partly conducted in high–risk subjects did not show statistically significant effects [5–11].

Studies on anti–*H. pylori* treatment considered reduction in gastric cancer incidence and mortality [15,17,20,21] or other outcomes eg, changes regarding precancerous lesions or *H. pylori* cure rate [12–14,16,18,19,22–26]. Eleven studies were conducted in China [13–17,19–22,24,26] and four studies in Colombia [12], Mexico [18], Turkey [23] and Iran [25]. The follow–up period ranged between 1–15 years. Nine studies used anti–*H. pylori* treatment [13,14,16–18,22,25,26] only, and six studies also considered other substances eg, vitamins, garlic, celecoxib [12,15,19–21,24]. The antibiotics regimens used for anti–*H. pylori* treatment varied across studies. Gastric cancer incidence and mortality tended to be reduced by 40–50% [15,17,20]. The study by Guo et al. treating high risk subjects with antibiotics and Chinese herb medicine suggested a statistically significant reduction in gastric cancer mortality (RR = 0.50, 95% CI 0.34–0.73) at 3 years follow–up [15]. The study by Ma et al. found a statistically significant reduction in gastric cancer incidence (odds ratio (OR) = 0.61, 95% CI 0.38 – 0.96) but not in mortality 15 years after anti–*H. pylori* treatment [20]. A subgroup analysis of the same study restricted to persons ≥55 years or older found a statistically significant reduction in gastric cancer incidence (OR = 0.36, 95% CI 0.17–0.79) and mortality (hazard ratio (HR) = 0.26, 95% CI 0.09–0.79) [21]. This study also found a reduction in gastric cancer incidence in persons with precancerous lesions (OR = 0.56, 95% CI 0.34 – 0.91)^1^, while the study by Wong et al. only found such an effect in persons without precancerous lesions (*P* = 0.02) [17]. Of the studies investigating the impact of anti–*H. pylori* treatment on precancerous lesions [12–14,16,25,29], four differentiated according to the type of lesions and found a regression mainly for atrophy while there was no or only a marginal effect regarding intestinal metaplasia [12,14,16,25]. Apart from anti–*H. pylori* treatment, one study using a factorial design suggested an effect regarding regression of precancerous lesions also for celecoxib (OR = 1.50, *P* = 0.067) [24].

Studies on gastric cancer screening considered reduction in gastric cancer mortality or intermediate outcomes (eg, gastric cancer detection rate) as outcomes. Observational studies suggested a statistically significant reduction of gastric cancer mortality for direct gastroscopy (odds ratio (OR) = 0.72, 95% CI 0.54 – 0.97) and X–ray (48–59%, *P* < 0.05) [30,34]. Screening with photofluorography was not found to reduce mortality from gastric cancer [29]. In the two studies on gastric cancer screening evaluating an occult blood bead detector (a device that is swallowed and then retrieved to detect occult blood in the stomach) the positivity rate ranged between 7–24% and the proportion of early stages of gastric cancers ranged between 45–85% [27,28]. With gastroscopy, proportion of gastric cancers detected at an early stage ranged between 60–100% [31–33].

### Liver cancer

Studies on liver cancer prevention could be classified into three main categories: hepatitis B virus (HBV) immunization (2 randomized trials, 2 cohort studies, 1 intervention trial and 2 cross–sectional studies), liver cancer screening (2 randomized trials and 1 screening pilot study) and supplementation with minerals (1 non–randomized and 2 randomized trials). **Table 2** summarizes information from these studies [35–58].

**Table 2:** Studies investigating strategies for prevention of liver cancer

Author(s), year	Preventive measure/screening tool	Country (region)	Study design	Study population	Outcome(s) under study	Results		
**HBV immunization:**								
Maupas et al., 1981 [35]	HBV immunization with: 10 µg HBVD; Vaccination regimen: 3 times at one month intervals	Senegal (Niakhar)	Cluster–randomized controlled trial	Sample size: n = 602; Sex distribution: 49% male; Age: range: 0–2 y	Reduction in incidence of HBsAg carrier state in susceptible children (seronegative and anti–HBc alone) at 1 y follow–up	85% (*P* < 0.005)		
Sun et al., 1986 [36]; Sun et al., 1991 [37]; Qu et al., 2014 [38]	HBV immunization with: 5 µg HBVD+HBIG; 5 µg HBVD; 2.5 µg HBVD+HBIG; 2.5 µg HBVD. Vaccination regimen: 3 times at 0, 1 and 6 months after birth	China (Qidong)	Cluster–randomized controlled trial	Sample size: n = 1703; Sex distribution: 50% male; Age: new–born infants	Reduction in incidence of HBsAg carrier state: determined at 1 y and 5 y follow–up	**1 y follow–up:**		**5 y follow–up:**
						5 µg HBVD+HBIG: 85%		5 µg HBVD+HBIG: 86%
						5 µg HBVD: 83%		5 µg HBVD: 80%
						2.5 µg HBVD+HBIG:65%		2.5 µg HBVD+HBIG: 62%
						2.5 µg HBVD: 85%		2.5 µg HBVD: 75%
	HBV immunization with: 5µg HBVD; Vaccination regimen: 3 times at 0, 1 and 6 months after birth; booster dose after 10 to 14 y	China (Qidong)	Cluster–randomized controlled trial	Sample size: n = 73 733; Sex distribution: 51% male; Age: new–born infants	Reduction in incidence of HBsAg carrier state: determined at 18 y follow–up; determined at 30 y follow–up. HR regarding liver cancer incidence rate at 30 y follow–up	78% (95% CI 75–80%)		
						72% (95% CI 68–75%)		
						HR: 0.16 (95% CI 0.03–0.77)		

Chotard et al., 1992 [39]; Fortuin et al., 1993 [40]; Viviani et al., 1999 [41]; Van der Sande et al., 2007 [42]; Peto et al., 2014 [43]	HBV immunization with: 10 µg HBVD; Vaccination regimen: 4 times at 0, 1, 4 and 9 months after birth	Gambia	Cohort study among vaccinated children combined with a cross–sectional survey among unvaccinated children	Sample size: n = 1000; Sex distribution: n.r.; Age: children that received HBV vaccine in infancy	Reduction in incidence of HBsAg carrier state determined at 3 y follow–up; determined at 4 y follow–up; determined at 9 y follow–up; determined at 15 y follow–up;	95% (95% CI n.r.)		
						94% (95% CI 84–98%)		
						94% (95% CI 84–98%)		
						97% (95% CI 91.5–100%)		
			Cross–sectional study	Sample size: n = 2670; Sex distribution: 44% male; Age: 17–21 y (birth years 1986–90)	Reduction in incidence of HBsAg carrier state determined at 21 y follow–up	94% (95% CI 77–99%)		
Whittle et al., 1991 [44]; Whittle et al., 1995 [45]; Whittle et al., 2002 [46]; Mendy et al., 2013 [47]	HBV immunization with varied HBVDs (2, 2.5, 5, 10 and 20 µg) and different vaccination regimens (3 or 4 times between 0–4 y)	Gambia (Keneba & Manduar)	Intervention trial	Sample size: n = 856 (continued recruitment); Sex distribution: n.r.; Age: range: 0–4 y	Reduction in incidence of HBsAg carrier state determined at 4 y follow–up; determined at 8 y follow–up; determined at 14 y follow–up; determined at 24 y follow–up	97% (95% CI 91.0–99.2%); 95% (95% CI 91.0–97.5%); 94% (95% CI 89–97%); 95% (95% CI 91.5–97.1%)		
Wichajarn et al., 2008 [48]	HBV immunization	Thailand (Khon Kaen)	Retrospective cohort study	Sample size: n = n.r. (newborns in Khon Kaen); Sex distribution: n.r.; Age: newborns	Age–standardized incidence rate of hepatocellular carcinoma in vaccinated vs non–vaccinated children aged 5–18 y	Non–vaccinated: 0.97 per million; vaccinated: 0.24 per million (*P* = 0.007)		
Shen et al. 2011 [49]	HBV immunization	China (Long An)	Cross–sectional study	Sample size: n = 4686; Sex distribution: 49% male; Age: range: 0.25–60 y, median: 34 y	Rate of HBsAg seroprevalence in subjects born before vs after start of the HBV vaccination programme (<20 y vs ≥20 y)	≥20 y: 10.5% (95% CI 9.4–11.7%); <20 y: 2.4% (95% CI 1.7–3.1%)		
Posuwan et al., 2016 [50]	HBV immunization	Thailand	Cross–sectional study	Sample size: n = 5964; Sex distribution: n.r.; Age: range: 0.5–60 y	Rate of HBsAg seroprevalence in subjects born before vs after start of the HBV vaccination programme (<22–24 y vs ≥22–24 y; exact cutoff depending on region)	≥22–24 y: 4.5%; <22–24 y: 0.6%; (*P* = 0.001)		
**Supplementation with minerals:**								
Yu et al., 1991 [51]; Yu et al., 1997 [52]	Daily supplementation of: selenium fortified salt in the general population. Duration of supplementation: 8 y	China (Qidong)	Placebo–controlled trial with intervention and control communities	Sample size: n = 130 471; Sex distribution: n.r.	Age–adjusted incidence of primary liver cancer in the intervention vs control group before and after the trial	Intervention group: before trial: 42/100 000; after trial: 27/100 000. Control group: no change		
	Daily supplementation of: selenized yeast tablets in HBsAg carriers. Duration of supplementation: 4 y	China (Qidong)	Randomized placebo–controlled trial	Sample size: n = 226 (HBsAg carriers); Sex distribution: n.r.; Age: range: 21–63 y	Incidence of primary liver cancer in the intervention vs the control group determined at the end of the trial	Intervention group: 0/113. Placebo group: 7/113		
Qu et al., 2007 [53]	Daily supplementation of: Factor A (retinol + zinc); Factor B (riboflavin + niacin); Factor C (vitamin C +molybdenum); Factor D (beta–carotene + vitamin E + selenium); Duration of supplementation: 5.25 y	China (Linxian)	Randomized trial with 2^4^factorial design	Sample size: n = 29 450; Sex distribution: 45% male; Age: range: 40–69 y	HR regarding reduction of liver cancer mortality determined at 15 y follow–up	Factor A: 0.86 (95% CI 0.62–1.18); Factor B: 0.86 (95% CI 0.62–1.18); Factor C: 0.84 (95% CI 0.61–1.16); Factor D: 0.81 (95% CI 0.59–1.12)		
**Early detection:**								
Yang et al., 1997 [54]; Zahng et al., 1999 [55]; Zahng et al., 2004 [56]	Biannual testing of serum alpha–fetoprotein	China (Shanghai)	Randomized controlled study	Sample size: n = 18816 (HBV infected or history of chronic hepatitis); Sex distribution: 63% male; Age: range: 35–55 y, mean: 53 y	Reduction in liver cancer mortality determined at 5 y follow–up (after 5–10 screening rounds)	RR: 0.63 (95% CI 0.41–0.98)		
Chen et al., 2003 [57]	Biannual testing of serum alpha–fetoprotein for 62 months	China (Qidong)	Randomized controlled study	Sample size: n = 5581 (HBsAg carriers); Sex distribution: 100% male; Age: range: 30–69 y, mean: 41 y	Liver cancer detection rate; Proportion of liver cancers detected at an early stage; Reduction in liver cancer mortality determined 6 y after start of the trial	7% (374/5581); 28% (67/240) (screen group); 4% (4/108) (control group); (*P* < 0.0001); 1138/100 0000 (screen group); 1114/100 000 (control group); (*P* = 0.86)		
Eltabbakh et al., 2015 [58]	Biannual testing of serum alpha–fetoprotein and ultrasonography of liver for at least 18 months	Egypt	Screening pilot study	Sample size: n = 1286 (patients with liver cirrhosis undergoing screening); Sex distribution: 35% male; Age: >18 y, mean: 51 y	Liver cancer detection rate; Proportion of liver cancers detected at an early stage in the screening cohort as compared to 155 symptomatic liver cancer patients	8% (102/1286); 89% (91/102) (screen–detected patients); 22% (35/155) (symptomatic patients) (*P* < 0.0001)		

Anti–HBc – hepatitis B core antibody, HBIG – Hepatitis B virus immune globulin, HBsAg – hepatitis B surface antigen, HBV – hepatitis B virus, HBVD – hepatitis B vaccine dose, HR – hazard ratio, n – number, n.r. – not reported, OR – odds ratio, y – year

Almost all HBV immunization studies assessed the effect of HBV vaccine in protecting against hepatitis B chronic carriage [35–47,49,50]. Two studies evaluated reduction in incidence of liver cancer in children and young adults [38,48]. All studies used plasma–derived hepatitis B vaccines except for one study [45] that used both plasma–derived and recombinant vaccines. Vaccination regimens varied widely in terms of dosage, number of vaccinations and time intervals between vaccinations. Reported follow–up period ranged between 1–30 years.

Reduction in the incidence of Hepatitis B surface Antigen (HBsAg) carrier state (ie, protection against chronic carriage) was consistently shown across studies, ranging between 65%–95% compared to non–vaccinated controls, and the effect remained stable during follow–up [35–47,49,50]. A cluster–randomized trial on HBV vaccination in newborns suggested a statistically significant reduction in liver cancer incidence in younger adults (hazard ratio HR = 0.16 (95% CI 0.03–0.77) and similarly, an incidence reduction was observed in a retrospective cohort study (*P* = 0.007) [38,48].

One study evaluated the effect of four combinations of various vitamins and minerals (eg, retinol and zinc or vitamin C and molybdenum etc.) regarding reduction of liver cancer mortality and did not show statistically significant effect [53]. Two studies conducted in China evaluated the effect of supplementation of selenium regarding liver cancer incidence in the general population and in HBsAg carriers. They suggested a reduction in age–adjusted incidence over time in the intervention group as compared to the control but it was not reported whether this was statistically significant [51,52].

Two studies investigated biannual testing of serum alpha fetoprotein to screen for liver cancer in high–risk subjects [54–57]. One study reported a statistically significant reduction in liver cancer mortality by 40% after 5 years of follow–up (RR = 0.63, 95% CI 0.41–0.98) [56]. The other study, however, did not suggest a statistically significant reduction in liver cancer mortality (*P* = 0.86). One study evaluated combination of biannual testing of serum alpha fetoprotein and liver ultrasonography to screen for liver cancer and reported that the proportion of liver cancers detected at an early stage was 90% [58].

### Colorectal cancer

With respect to CRC prevention, 18 screening studies conducted mainly in average–risk subjects were identified. They investigated colonoscopy (6 cross–sectional diagnostic studies and 1 screening pilot study), rectoscopy (1 cross–sectional diagnostic study and 1 cohort study with external control group), and fecal occult blood testing (2 cross–sectional diagnostic studies and 7 screening pilot studies). **Table 3** summarizes information about these studies [59–76].

**Table 3:** Studies investigating strategies for prevention of colorectal cancer

Author(s), year	Preventive measure/screening tool	Country (region)	Study design	Study population	Outcome(s) under study	Results					
**Colonoscopy screening**											
Zheng et al., 1991 [59]	Rectoscopy (alone or in combination with fecal occult blood testing)	China (Jiashan)	Cross–sectional diagnostic study	Sample size: n = 26 171; Sex distribution: n.r.; Age: ≥30 y	Polyp detection rate; Rectal cancer detection rate; Proportion of rectal cancers detected at an early stage	3% (899/26 171); 0.05% (15/26 171); 53% (8/15)					
Zheng et al., 2002 [60]	Proctoscopy. Endoscopic follow–up of individuals with removed precursor lesions every 2–5 y	China (Haining)	Cohort study with external control group	Sample size: n = 4072; Sex distribution: 64% male; Age: range: 30–70 y; mean: 50 y	Standardized incidence ratio and standardized mortality ratio determined at 20 y follow–up	Rectal cancer Standardized incidence ratio: 0.69; Standardized mortality ratio: 0.82. Colon cancer: no effect was observed.					
Wan et al., 2002 [61]	Colonoscopy	China	Cross–sectional diagnostic study	Sample size: n = 2196 (74% were asymptomatic); Sex distribution: 94% male; Age: range: 60–90 y; average: 70 y	Polyp detection rate; CRC detection rate; Proportion of CRCs detected at an early stage	62% (1364/2196); 2% (52/2196); 37% (19/52)					
Croitoru et al., 2010 [62]	Colonoscopy	Romania (Suceava & Iasi)	Cross–sectional diagnostic study	Sample size: n = 102; (all asymptomatic and with at least one first–degree relative with CRC); Sex distribution: 57% male; Age: range: 36–72 y; mean: 52 y	Participation rate; CRC detection rate; Proportion of CRCs detected at an early stage	47% (102/216); 2% (2/102); 50% (1/2)					
Arafa et al., 2011 [63]	Colonoscopy	Jordan (Hashemite)	Cross–sectional diagnostic study	Sample size: n = 95 (symptomatic first degree relatives of CRC patients); Sex distribution: 61% male; Age: range: 40–75 y, mean: 53 y	Participation rate; Polyp detection rate; CRC detection rate; Proportion of CRCs detected at an early stage	62% (95/153); 11%(10/95) 2% (2/95); 100% (2/2)					
Aswakul et al., 2012 [64]	Colonoscopy	Thailand	Cross–sectional diagnostic study	Sample size: n = 1594 (asymptomatic average and high risk individuals); Sex distribution: 45% male; Age: mean: 58 y	Adenoma detection rate; Advanced adenoma detection rate; CRC detection rate	16% (263/1954); 3% (43/1594); 0.6% (10/1594)					
Ionescu et al., 2015 [65]	Colonoscopy	Romania (Bucharest)	Cross–sectional diagnostic study	Sample size: n = 1087 (average risk individuals); Sex distribution: 47% male; Age: range: 23–97 y, mean: 58 y	Adenoma detection rate; Advanced adenoma detection rate; CRC detection rate	13% (228/1807); 6% (110/1807); 3% (61/1807)					
Panic et al., 2015 [66]	Colonoscopy	Montenegro	Cross–sectional diagnostic study	Sample size: n = 540 (first–degree relatives of CRC patients); Sex distribution: 41% male; Age: >40 y or 10 y before index case age	Participation rate; Adenoma detection rate; Advanced adenoma detection rate; CRC detection rate	76% (540/710); 28% (151/540); 11% (58/540); 6% (31/540)					
	FIT	Montenegro	Cross–sectional diagnostic study	Sample size: n = 920 (average risk individuals); Sex distribution: 51% male; Age: range: 50–74 y	Participation rate; Adenoma detection rate; Advanced adenoma detection rate; CRC detection rate	33% (920/2760); 3% (26/920); 2% (19/920); 1% (5/920)					
Siripongpreeda et al., 2016 [67]	Colonoscopy	Thailand	Screening pilot study	Sample size: n = 1404 (average–risk); Sex distribution: 31% male; Age: mean: 57 y	Participation rate; Adenoma detection rate; Advanced adenoma detection rate; CRC detection rate; Proportion of CRCs detected at an early stage	87% (1404/1612); 18% (256/1404); 7% (98/1404); 1% (18/1404); 89% (16/18)					
Li et al. 2003 [68]	Sequential FOBT (guaiac FOBT followed by FIT)*	China (Beijing)	Screening pilot study	Sample size: n = 19852; Sex distribution: 51% male; Age: mean: 50 y	Participation rate; Positivity rate; Polyp detection rate; CRC detection rate; Proportion of CRCs detected at an early stage	74% (19852/26827) 3% (501/19852) 1% (188/19852) 0.06%(12/19852) 92% (11/12)					
Li et al. 2006 [69]	Guaiac FOBT, FIT, sequential FOBT (comparative evaluation)*	China (Beijing)	Cross–sectional diagnostic study	Sample size: n = 323 (patients referred for colonoscopy); Sex distribution: 57% male; Age: range: 18–68 y, mean: 53 y	Sensitivity and specificity of guaiac FOBT, FIT and sequential FOBT regarding CRC	**Test**		**Two–sample**		**Three–sample**	
						FIT Guaiac FOBT Sequential FOBT FIT Guaiac FOBT Sequential FOBT		Sensitivity 88% 78% 76% Specificity 96% 89% 99%		Specificity 96% 96% 94% Specificity 89% 76% 94%	
Fenocchi et al., 2006 [70]	FIT	Uruguay (Montevideo)	Screening pilot study	Sample size: n = 10 573 (average–risk); Sex distribution: 31% male; Age: mean: 61 y	Participation rate; Positivity rate; Proportion of test positives undergoing colonoscopic follow–up; CRC detection rate; Proportion of CRCs detected at an early stage	90% (10 573/11 734); 11% (1,170/10 573); 75% (879/1170); 1% (101/10 573); 47%(47/101)					
Yang et al., 2011 [71]	FIT	China (Shanghai)	Screening pilot study	Sample size: n = 5919; Sex distribution: 55% male; Age: mean: 55 y	Positivity rate; Proportion of test positives undergoing FIT follow–up; CRC detection rate; Proportion of CRC detected at an early stage; Adenoma detection rate	5% (314/5919); 84% (264/314); 0.2% (16/5919); 94% (15/16); 1% (94/5919)					
Khuhamprema et al., 2014 [72]	FIT	Thailand (Lampang)	Screening pilot study	Sample size: n = 127 301; Sex distribution: 46% male; Age: range: 50–65 y	Participation rate; Positivity rate; Proportion of test positives undergoing colonoscopic follow–up; CRC detection rate; proportion of CRC detected at an early stage; Adenoma detection rate	63% (80 012/127 301); 1% (873/80 012); 72% (627/873); 4% (23/627); 61% (14/23); 30% (187/627)					
Dimova et al., 2015 [73]	FIT	Bulgaria	Screening pilot study	Sample size: n = 600 (average–risk); Sex distribution: 45% male; Age: mean: 61 y	Participation rate; Positivity rate; Proportion of test positives with information on colonoscopy; CRC detection rate	79% (473/600); 8% (40/473); 75% (30/40); 0.6% (3/473)					
Bankovic et al., 2016 [74]	FIT	Serbia	Screening pilot study	Sample size: n = 99 592; Sex distribution: n.r.; Age: range:50–74 y	Participation rate; Positivity rate; Proportion of test positives undergoing colonoscopic follow–up; CRC detection rate; Adenoma detection rate	62% (62252/99592); 6% (3690/62252); 42% (1554/3690); 8% (129/1554); 38% (586/1554)					
Zheng et al., 2003 [75]	Step 1: Risk stratification based on clinical score combined with FIT result; Step 2: Flexible sigmoidoscopy	China (Jiashan)	Cluster–randomized screening pilot study	Sample size: n = 62 677 (average –risk); Sex distribution: 51% male; Age: ≥30 y	Positivity rate; Polyp detection rate; CRC detection rate; Proportion of CRC detected at an early stage; Mortality and incidence rate of CRC in the screening vs control group at 8 y follow–up	7% (4299/62677); 0.5% (331/62677); 0.03% (21/62677); 71% (15/21)					
						**Mortality rate:**					
						208/100 000 (95% CI 196–218/100 000) (screening group)					
						244/100 000 (95% CI 233–255/100 000) (control group)					
						**Incidence rate:**					
						395/100 000 (95% CI 381–410/100 000) (screening group)					
						401/100 000 (95% CI 386–411/100 000) (control group)					
Aniwan et al., 2015 [76]	Step 1: Risk stratification based on clinical score combined with FIT result; Step 2: Colonoscopy	Thailand (Bankok)	Cross–sectional diagnostic study	Sample size: n = 948 (average risk); Sex distribution: 35% male; Age: range: 50–75 y, mean: 61 y	Polyp and CRC detection rate	**Category**	**Non–advanced neoplasia**		**Advanced neoplasia**		**CRC**
						High risk score and positive FIT (n = 84)	44%		37%		5%
						High risk score and negative FIT (n = 173)	30%		12%		1%
						Moderate risk score and positive FIT (n = 192)	27%		12%		2%
						Moderate risk score and negative FIT (n = 499)	23%		6%		0%

CRC – colorectal cancer, FIT – fecal immunochemical testing for hemoglobin, FOBT – fecal occult blood testing, n – number, n.r. – not reported, y – year

*Sequential FOBT was called a sequential method that combined guaiac FOBT and FIT, ie, guaiac FOBT was performed first and FIT was only performed if the guaiac FOBT was positive. The result was interpreted as positive if both tests were positive.

In colonoscopy studies, CRC detection rates were between 0.5–6% [61–67]. One study assessed proctoscopy with regular endoscopic follow–up of persons in whom precursor were removed. It suggested a reduction in rectal cancer incidence and mortality of 31% and 18% at 20 years follow–up, respectively, when compared to an external control group [60]. Three studies reported a participation rate above 40% for colonoscopy in first–degree relatives of CRC patients [62,63,66].

In studies on fecal occult blood testing, CRC detection rates were directly associated with positivity rates in five of six studies [68,70–74]. The lowest CRC detection rate (0.06%) was reported from a study on sequential fecal occult blood testing [68]. The highest positivity rates were reported from studies using fecal immunochemical testing for hemoglobin (FIT) [70–74]. The proportion of early stages among detected CRCs ranged between 47–94% [68,70–72]. Two studies investigated the potential of risk stratification using a clinical risk score combined with FIT [75.76]. One of these studies found a five–fold higher rate of advanced neoplasia among those with both a high–risk score and a positive FIT result as compared to those with a moderate–risk score and a negative FIT result [76]. The other study, a cluster–randomized study, suggested a reduction in CRC mortality after 8 years follow–up [75]. Five studies reported participation rates for fecal occult blood testing above 60% [68,70,72–74].

## DISCUSSION

Our systematic review identified a wide range of studies evaluating strategies for prevention of GICs in developing countries, including follow–up reports up to 30 years. Studies on gastric and liver cancer prevention showing promising results only after long–term follow–up illustrate the particular challenge of generating evidence in cancer prevention.

The development and evaluation of strategies for cancer prevention is a long–lasting process. The duration of this process is amongst others determined by the natural history of the disease, ie, the time that it takes until risk factors, precursor lesions or preclinical cancer stages would have impacted on the disease incidence or mortality if they had remained unchanged or untreated. If the interruption of the natural history takes place at an early phase in life, the time lag until a potential effect is measurable at the population level is further prolonged.

The studies included in our review on anti–*H. pylori* treatment to prevent gastric cancer can be considered as an example where the process of developing a preventive strategy takes long. With a follow–up time of 7.5 years after *H. pylori* treatment the randomized controlled trial by Wong et al. found an incidence reduction of 40% for gastric cancer but this effect did not reach statistical significance [17]. With a follow–up time of 15 years Ma et al. found a similar effect that was statistically significant. Regarding the effectiveness of anti–*H. pylori* treatment in persons who already have precancerous lesions the evidence is still not conclusive. Some but not all studies found a regression of precancerous lesions after *H. pylori* treatment. One study reported a preventive effect only in subgroup analysis restricted to persons without precancerous lesions [17]. A recent study by Li et al. analyzing 15–year follow–up data suggested a reduction in the incidence of gastric cancer by 40% among subjects with intestinal metaplasia and dysplasia treated against *H. pylori* at baseline [21]. As for vaccination programs, *H. pylori* treatment is a preventive measure that can be completed within a narrow time window and does not need to be repeated on a regular basis, which is an important aspect in view of large–scale feasibility and acceptance.

The studies included in our review on HBV vaccination published in 1981 and later can be considered as an example where the process to develop a preventive strategy that is widely accepted and applied was relatively short. Robust evidence showed that vaccine efficacy against chronic carriage of HBV was as high as 65–95% across studies and remained stable with time. Since 1992 the World Health Organization (WHO) has recommended that all infants receive the HBV vaccine as soon as possible after birth. As of 2013, 183 WHO member states have included HBV vaccination in their preventive programs [77]. While the various beneficial effects of these programs regarding HBV–related diseases are out of question, it will take more time to see their full effect on incidence and mortality of liver cancer, particularly in adults. The study by Wichajarn et al. [48] already observed an incidence reduction for hepatocellular carcinoma among children in Thailand, which confirms earlier reports from Taiwan [78]. A study from China reported an incidence reduction of 84% in a study population that reached early adulthood [38]. This also confirms earlier reports from Taiwan showing that the preventive effect of HBV vaccination extends from childhood to early adulthood [79].

Studies included in our review on the prevention of gastric and liver cancer by supplementation of vitamins or minerals in developing countries give the impression that there is a lack of effectiveness or not enough evidence yet to justify their translation into a preventive program. Most studies did not show an effect or were difficult to interpret. This is in line with results of supplementation trials conducted in Western countries that did not show beneficial effects either, such as the Selenium and Vitamin E Cancer Prevention Trial [80]. Apart from the lack of effectiveness, it is questionable whether preventive measures that require long–term and regular use of supplements would prove suitable for developing countries. Food or soil fortification may have more potential in terms of practicality, but of course this would become relevant only for measures with proven effectiveness and safety [81].

Practicality is also an important aspect to be discussed in the context of cancer screening in developing countries. The screening strategies included in this review, mainly focused on colorectal cancer, which is less amenable to primary prevention through risk factor modification as compared to gastric and liver cancer. Implementing population–based screening programs may be challenging in developing countries in view of resource constraints (eg, infrastructure, availability of equipment and trained personnel, costs). This notion is supported by the fact that substantial proportion of screening studies has been conducted in China that has more resources than many other developing countries. While most screening studies were limited to intermediate endpoints (eg, detection rates), Zahng et al. reported a reduction in liver cancer mortality by 40% for biannual serum alpha–fetoprotein testing in high risk groups (HBV infected or history of chronic hepatitis) after 5–10 screening rounds [56]. Targeting screening at high–risk groups rather than at average–risk persons may generally be a more doable approach for developing countries that seems worthwhile to be further explored, eg, also for colorectal cancer. To ensure practicality of such strategies, identification of risk groups needs to be based on easily obtainable information (eg, family history, lifestyle or basic clinical factors). This was exemplified by two studies that used a clinical risk score combined with FIT to identify risk groups that benefit most from colonoscopy [75,76]. However, when estimating the effectiveness of potential screening strategies in developing countries, the treatability of early cancer stages also requires consideration. In industrialized countries, an important argument in favor of screening is the better prognosis for early vs late stages, but this may not hold true in developing countries if, for example, surgical treatment options are limited [82].

To the best of our knowledge, there is no similar review that systematically summarizes studies on the prevention of gastric, liver and colorectal cancer in developing countries including long–term follow–up reports on these studies. Considerable variation in prevalence of various cancers between low– and high–resource countries, extensive differences in operational settings and possibly also in compliance rates warrant to put a focus on strategies for cancer prevention specifically in developing countries. There are also limitations that should be noted. First, our search was restricted to papers published in English and we did not optimize our search for specific sub–questions such as HBsAg carrier state nor did we include the aspect of cost–effectiveness. Second, incomplete reporting of relevant information in original articles partly limited interpretability of the studies. Third, our review provides a descriptive summary of studies, while meta–analyses would have been beyond its scope. However, we consider our review an important pre–work that will facilitate the planning and conduct of such meta–analyses, particularly by providing information regarding heterogeneity between studies (in terms of the study designs, the target populations, the interventions, the follow–up periods, the outcomes etc.). Fourth, as for any review, we cannot rule out that publication bias has led to overestimating the beneficial effect of preventive measures. In the interpretation of this review it should also be noted that almost half of the studies were conducted in China, but developing countries considerably vary in their Human Development Index and aspects of effectiveness and feasibility may not be similar across countries.

In conclusion, there were a number of studies on gastric and liver cancer prevention in developing countries showing promising results after long–term follow–up. Important next steps include pooled meta–analyses as far as possible given the heterogeneity between studies as well as implementation research.
