# Supplementary material for: Strategies for prevention of gastrointestinal cancers in developing countries: a systematic review
Source: J Glob Health. 2017 Nov 17;7(2):020405. doi: 10.7189/jogh.07.020405 (PMC5718709; doi:10.7189/jogh.07.020405)
Supplement: Online Supplementary Document [file jogh-07-020405-s001.pdf]

Online Supplementary Document

Shams et al. Strategies for prevention of gastrointestinal cancers in developing countries: a systematic review

J Glob Health 2017;7:020405

Appendix S1. Search Strategy

A. **PubMed Search String**
   MeSH Term Search:

Search name	Search query
A1	Digestive System Neoplasm[MeSH] OR Gastrointestinal Neoplasm [MeSH]
A2	Developing country [MeSH] OR Bahrain [MeSH] OR Bahamas [MeSH] OR Belarus [MeSH] OR Uruguay [MeSH] OR Montenegro [MeSH] OR Palau [MeSH] OR Kuwait [MeSH] OR Russian Federation [MeSH] OR Romania [MeSH] OR Bulgaria [MeSH] OR Saudi Arabia [MeSH] OR Cuba [MeSH] OR Panama [MeSH] OR Mexico [MeSH] OR Costa Rica[MeSH] OR Grenada [MeSH] OR Libya [MeSH] OR Malaysia [MeSH] OR Serbia [MeSH] OR Antigua [MeSH] OR Trinidad [MeSH] OR Kazakhstan [MeSH] OR Albania [MeSH] OR Venezuela [MeSH] OR Dominica [MeSH] OR Georgia [MeSH] OR Lebanon [MeSH] OR Saint Kitts and Nevis [MeSH] OR Iran [MeSH] OR Peru [MeSH] OR Macedonia [MeSH] OR Ukraine [MeSH] OR Mauritius [MeSH] OR Bosnia and Herzegovinian [MeSH] OR Azerbaijan [MeSH] OR Saint Vincent [MeSH] OR Oman [MeSH] OR Brazil [MeSH] OR Jamaica [MeSH] OR [MeSH] Armenia [MeSH] OR Saint Lucia [MeSH] OR Ecuador [MeSH] OR Turkey [MeSH] OR Colombia [MeSH] OR Sri Lanka [MeSH] OR Algeria [MeSH] OR Tunisia [MeSH] OR Tonga [MeSH] OR Belize [MeSH] OR Dominican Republic [MeSH] OR Fiji [MeSH] OR Samoa [MeSH] OR Jordan[MeSH] OR China [MeSH] OR Turkmenistan [MeSH] OR Thailand [MeSH] OR Maldives [MeSH] OR Suriname [MeSH] OR Gabon [MeSH] OR El Salvador [MeSH] OR Bolivia [MeSH] OR Mongolia [MeSH] OR Palestine [MeSH] OR Paraguay [MeSH] OR Egypt [MeSH] OR Moldova [MeSH] OR Philippines [MeSH] OR Uzbekistan [MeSH] OR Syria [MeSH] OR Micronesia [MeSH] OR Guyana [MeSH] OR [MeSH] Botswana [MeSH] OR [MeSH] Honduras [MeSH] OR Indonesia [MeSH] OR Kiribati [MeSH] OR South Africa [MeSH] OR Vanuatu [MeSH] OR Kyrgyzstan [MeSH] OR Tajikistan [MeSH] OR Viet Nam [MeSH] OR Namibia [MeSH] OR Nicaragua [MeSH] OR Morocco [MeSH] OR Iraq [MeSH] OR Cape Verde [MeSH] OR Timor-Leste [MeSH] OR Ghana [MeSH] OR Guinea [MeSH] OR India [MeSH] OR Cambodia [MeSH] OR Lao

	[MeSH] OR Bhutan [MeSH] OR Swaziland [MeSH] OR Congo [MeSH] OR Solomon Islands [MeSH] OR Sao Tome [MeSH] OR Kenya [MeSH] OR Bangladesh [MeSH] OR Pakistan [MeSH] OR Angola [MeSH] OR Myanmar [MeSH] OR Cameroon [MeSH] OR Madagascar [MeSH] OR Tanzania [MeSH] OR Nigeria [MeSH] OR Senegal [MeSH] OR Mauritania [MeSH] OR Papua new Guinea [MeSH] OR Nepal [MeSH] OR Lesotho[MeSH] OR Togo [MeSH] OR Yemen [MeSH] OR Haiti [MeSH] OR Uganda [MeSH] OR Zambia [MeSH] OR Djibouti [MeSH] OR Gambia [MeSH] OR Benin [MeSH] OR Rwanda [MeSH] OR Cote dIvoire [MeSH] OR Comoros [MeSH] OR Malawi [MeSH] OR Sudan [MeSH] OR Zimbabwe [MeSH] OR Ethiopia [MeSH] OR Liberia [MeSH] OR Afghanistan [MeSH] OR Guinea Bissau [MeSH] OR Sierra Leone [MeSH] OR Burundi [MeSH] OR Senegal Guinea [MeSH] OR Central African Republic [MeSH] OR Eritrea [MeSH] OR Mali [MeSH] OR Burkina Faso [MeSH] OR Chad [MeSH] OR Mozambique [MeSH] OR Congo [MeSH] OR Niger [MeSH]
A3	Prevention [MeSH]OR Early Detection of Cancer[MeSH]
A1 AND A2 AND A3	

For the additional free text (Keyword) search, the following strings were used for A1 and A3

**Search name**	**Search query**
A1	(gastric tumor*[Title/Abstract] OR stomach tumor*[Title/Abstract] OR gastric malignancy*[Title/Abstract] OR stomach malignancy OR liver tumor*[Title/Abstract] OR liver malignancy *[Title/Abstract] OR large bowel neoplasm* [Title/Abstract] OR large bowel tumor *[Title/Abstract] OR large bowel malignancy*[Title/Abstract] OR colon neoplasm *[Title/Abstract] OR colon tumor *[Title/Abstract] OR colon malignancy *[Title/Abstract] OR rectal neoplasm*[Title/Abstract] OR rectal tumor *[Title/Abstract] OR rectal malignancy *[Title/Abstract])
A3	(Cancer screening *[Title/Abstract] OR primary prevention*[Title/Abstract] OR secondary prevention*[Title/Abstract] AND (neoplasm *[Title/Abstract])

B. Global Index Medicus Search

Due to very basic indexing processes in the Global Index Medicus database, we were recommended by experts with experience in Global Index Medicus to use very general terms (e.g. digestive system

cancer, prevention) and then determine if there are hits related to developing countries in the search results.

C. Web of Science Search String

**Search name**	**Search query**
C1	“gastric neoplasm” OR “stomach neoplasm” OR “gastric cancer” OR “stomach cancer” OR “gastric neoplasms” OR “stomach neoplasms” OR “gastric cancers” OR “stomach cancers” OR “cancer of the stomach” OR “neoplasm of the stomach” OR “stomach tumor” OR “stomach tumors” OR “gastric tumor” OR “gastric tumors” OR “hepatic neoplasms” OR “hepatic neoplasm” OR “liver neoplasm” OR “liver neoplasms” OR “cancer of liver” OR “cancers of liver” OR “hepatocellular cancer” OR “hepatocellular carcinoma” OR “hepatocellular cancers” OR “hepatocellular carcinomas” OR “liver cancer” OR “liver cancers” OR “liver tumor” OR “liver tumors” OR “hepatocellular tumor” OR “hepatocellular tumors” OR “hepatic tumor” OR “hepatic tumors” OR “colorectal neoplasm” OR “colorectal neoplasms” OR “colorectal cancer” OR “colorectal cancers” OR “colorectal tumor” OR “colorectal tumors” OR “colorectal carcinoma” OR “colorectal carcinomas” OR “large bowel neoplasm” OR “large bowel neoplasms” OR “large bowel cancer” OR “large bowel cancers” OR “large bowel tumor” OR “large bowel tumors” OR “rectal cancer” OR “rectal cancers” OR “rectal neoplasm” OR “rectal neoplasms” OR “rectal tumor” OR “rectal tumors” OR “cancer of rectum” OR “cancers of rectum” OR “colon cancer” OR “colon cancers” OR “colon neoplasm” OR “colon neoplasms” OR “colon tumor” OR “colon tumors” OR “colon neoplasm” OR “colon neoplasms”

C2	“Bahrain” OR “Bahamas” OR “Belarus” OR “Uruguay” OR “Montenegro” OR “Palau” OR “Kuwait” OR “Russian Federation” OR “Romania” OR “Bulgaria” OR “Saudi Arabia” OR “Cuba” OR “Panama” OR “Mexico” OR “Costa Rica” OR “Grenada” OR “Libya” OR “Malaysia” OR “Serbia” OR “Antigua” OR “Trinidad” OR “Kazakhstan” OR “Albania” OR “Venezuela” OR “Dominica” OR “Georgia” OR “Lebanon” OR “Saint Kitts and Nevis” OR “Iran” OR “Peru” OR “Macedonia” OR “Ukraine” OR “Mauritius” OR “Bosnia and Herzegovinian” OR “Azerbaijan” OR “Saint Vincent” OR “Oman” OR “Brazil” OR “Jamaica” OR “Armenia” OR “Saint Lucia” OR “Ecuador” OR “Turkey” OR “Colombia” OR “Sri Lanka” OR “Algeria” OR “Tunisia” OR “Tonga” OR “Belize” OR “Dominican Republic” OR “Fiji” OR “Samoa” OR “Jordan” OR “China” OR “Turkmenistan” OR “Thailand” OR “Maldives” OR “Suriname” OR “Gabon” OR “El Salvador” OR “Bolivia” OR “Mongolia” OR “Palestine” OR “Paraguay” OR “Egypt” OR “Moldova” OR “Philippines” OR “Uzbekistan” OR “Syria” OR “Micronesia” OR “Guyana” OR “Botswana” OR “Honduras” OR “Indonesia” OR “Kiribati” OR “South Africa” OR “Vanuatu ” OR “Kyrgyzstan” OR “Tajikistan” OR “Viet Nam” OR “Namibia” OR “Nicaragua” OR “Morocco” OR “Iraq” OR “Cape Verde” OR “Timor-Leste” OR “Ghana” OR “Guinea” OR “India” OR “Cambodia” OR “Lao” OR “Bhutan” OR “Swaziland” OR “Congo” OR “Solomon Islands” OR “Sao Tome” OR “Kenya” OR “Bangladesh” OR “Pakistan” OR “Angola” OR “Myanmar” OR “Cameroon” OR “Madagascar” OR “Tanzania” OR “Nigeria” OR “Senegal” OR “Mauritania” OR “Papua new Guinea” OR “Nepal” OR “Lesotho” OR “Togo” OR “Yemen” OR “Haiti” OR “Uganda” OR “Zambia” OR “Djibouti” OR “Gambia” OR “Benin” OR “Rwanda” OR “Cote dIvoire” OR “Comoros” OR “Malawi” OR “Sudan” OR “Zimbabwe” OR “Ethiopia” OR “Liberia” OR “Afghanistan” OR “Guinea Bissau” OR “Sierra Leone” OR “Burundi” OR “Senegal Guinea” OR “Central African Republic” OR “Eritrea” OR “Mali” OR “Burkina Faso” OR “Chad” OR “Mozambique” OR “Congo” OR “Niger”
C3	“prevention” OR “early detection” OR “screening” OR “primary prevention” OR “secondary prevention” OR “chemoprevention” OR “vaccination” OR “gastroscopy” OR “vitamin and mineral supplementation” OR “H.pylori eradication” OR “occult blood bead detector” OR “Photofluorography” OR “immunization” OR “colonoscopy” OR “fecal occult blood test” OR “fecal immunochemical testing for haemoglobin”
C1 AND C2 AND C3	

Appendix S2. Name of Developing Countries used in Search Strings

No	Developing Country
1	Bahrain
2	Bahamas
3	Belarus
4	Uruguay
5	Montenegro
6	Palau
7	Kuwait
8	Russian Federation
9	Romania
10	Bulgaria
11	Saudi Arabia
12	Cuba
13	Panama
14	Mexico
15	Costa Rica
16	Grenada
17	Libya
18	Malaysia
19	Serbia
20	Antigua
21	Trinidad
22	Kazakhstan
23	Albania
24	Venezuela

25	Dominica
26	Georgia
27	Lebanon
28	Saint Kitts and Nevis
29	Iran
30	Peru
31	Macedonia
32	Ukraine
33	Mauritius
34	Bosnia and Herzegovinian
35	Azerbaijan
36	Saint Vincent
37	Oman
38	Brazil
39	Jamaica
40	Armenia
41	Saint Lucia
42	Ecuador
43	Turkey
44	Colombia
45	Sri Lanka
46	Algeria
47	Tunisia
48	Tonga
49	Belize
50	Dominican Republic

51	Fiji
52	Samoa
53	Jordan
54	China
55	Turkmenistan
56	Thailand
57	Maldives
58	Suriname
59	Gabon
60	El Salvador
61	Bolivia
62	Mongolia
63	Palestine
64	Paraguay
65	Egypt
66	Moldova
67	Philippines
68	Uzbekistan
69	Syria
70	Micronesia
71	Guyana
72	Botswana
73	Honduras
74	Indonesia
75	Kiribati
76	South Africa

77	Vanuatu
78	Kyrgyzstan
79	Tajikistan
80	Viet Nam
81	Namibia
82	Nicaragua
83	Morocco
84	Iraq
85	Cape Verde
86	Guatemala
87	Timor-Leste
88	Ghana
89	Guinea
90	India
91	Cambodia
92	Lao
93	Bhutan
94	Swaziland
95	Congo
96	Solomon Islands
97	Sao Tome
98	Kenya
99	Bangladesh
100	Pakistan
101	Angola
102	Myanmar

103	Cameroon
104	Madagascar
105	Tanzania
106	Nigeria
107	Senegal
108	Mauritania
109	Papua New Guinea
110	Nepal
111	Lesotho
112	Togo
113	Yemen
114	Haiti
115	Uganda
116	Zambia
117	Djibouti
118	Gambia
119	Benin
120	Rwanda
121	Cote dIvoire
122	Comoros
123	Malawi
124	Sudan
125	Zimbabwe
126	Ethiopia
127	Liberia
128	Afghanistan

129	Guinea Bissau
130	Sierra Leone
131	Burundi
132	Senegal Guinea
133	Central African Republic
134	Eritrea
135	Mali
136	Burkina Faso
137	Chad
138	Mozambique
139	Congo
140	Niger

Appendix S3. PRISMA checklist

Section/topic	#	Checklist item	Reported on page #
**TITLE**			
Title	1	Identify the report as a systematic review, meta-analysis, or both.	1
**ABSTRACT**			
Structured summary	2	Provide a structured summary including, as applicable: background; objectives; data sources; study eligibility criteria, participants, and interventions; study appraisal and synthesis methods; results; limitations; conclusions and implications of key findings; systematic review registration number.	2
**INTRODUCTION**			
Rationale	3	Describe the rationale for the review in the context of what is already known.	3-4
Objectives	4	Provide an explicit statement of questions being addressed with reference to participants, interventions, comparisons, outcomes, and study design (PICOS).	4
**METHODS**			
Protocol and registration	5	Indicate if a review protocol exists, if and where it can be accessed (e.g., Web address), and, if available, provide registration information including registration number.	None
Eligibility criteria	6	Specify study characteristics (e.g., PICOS, length of follow-up) and report characteristics (e.g., years considered, language, publication status) used as criteria for eligibility, giving rationale.	4
Information sources	7	Describe all information sources (e.g., databases with dates of coverage, contact with study authors to identify additional studies) in the search and date last searched.	4-5
Search	8	Present full electronic search strategy for at least one database, including any limits used, such that it could be repeated.	4, Supplement 1
Study selection	9	State the process for selecting studies (i.e., screening, eligibility, included in systematic review, and, if applicable, included in the meta-analysis).	4-5
Data collection process	10	Describe method of data extraction from reports (e.g., piloted forms, independently, in duplicate) and any processes for obtaining and confirming data from investigators.	5
Data items	11	List and define all variables for which data were sought (e.g., PICOS, funding sources) and any assumptions and simplifications made.	4-5

Risk of bias in individual studies	12	Describe methods used for assessing risk of bias of individual studies (including specification of whether this was done at the study or outcome level), and how this information is to be used in any data synthesis.	27-40 ("Study design" column in tables)
Summary measures	13	State the principal summary measures (e.g., risk ratio, difference in means).	27-40 "Outcome under Study" column in tables)
Synthesis of results	14	Describe the methods of handling data and combining results of studies, if done, including measures of consistency (e.g., I^2) for each meta-analysis.	5

Section/topic	#	Checklist item	Reported on page #
Risk of bias across studies	15	Specify any assessment of risk of bias that may affect the cumulative evidence (e.g., publication bias, selective reporting within studies).	27-40 ("Study design" column in tables)
Additional analyses	16	Describe methods of additional analyses (e.g., sensitivity or subgroup analyses, meta-regression), if done, indicating which were pre-specified.	Not applicable
**RESULTS**			
Study selection	17	Give numbers of studies screened, assessed for eligibility, and included in the review, with reasons for exclusions at each stage, ideally with a flow diagram.	41
Study characteristics	18	For each study, present characteristics for which data were extracted (e.g., study size, PICOS, follow-up period) and provide the citations.	27-40 (Results tables)
Risk of bias within studies	19	Present data on risk of bias of each study and, if available, any outcome level assessment (see item 12).	27-40

			("Study design" column in tables)
Results of individual studies	20	For all outcomes considered (benefits or harms), present, for each study: (a) simple summary data for each intervention group (b) effect estimates and confidence intervals, ideally with a forest plot.	27-40 ("Results" column in tables)
Synthesis of results	21	Present results of each meta-analysis done, including confidence intervals and measures of consistency.	Not applicable
Risk of bias across studies	22	Present results of any assessment of risk of bias across studies (see Item 15).	27-40 ("Study design" column in tables)
Additional analysis	23	Give results of additional analyses, if done (e.g., sensitivity or subgroup analyses, meta-regression [see Item 16]).	Not applicable
**DISCUSSION**			
Summary of evidence	24	Summarize the main findings including the strength of evidence for each main outcome; consider their relevance to key groups (e.g., healthcare providers, users, and policy makers).	11-17
Limitations	25	Discuss limitations at study and outcome level (e.g., risk of bias), and at review-level (e.g., incomplete retrieval of identified research, reporting bias).	16
Conclusions	26	Provide a general interpretation of the results in the context of other evidence, and implications for future research.	17
**FUNDING**			
Funding	27	Describe sources of funding for the systematic review and other support (e.g., supply of data); role of funders for the systematic review.	18

From: Moher D, Liberati A, Tetzlaff J, Altman DG, The PRISMA Group (2009). Preferred Reporting Items for Systematic Reviews and Meta-Analyses: The PRISMA Statement. PLoS Med 6(7): e1000097. doi:10.1371/journal.pmed1000097

For more information, visit: **www.prisma-statement.org**.
